# Tweety homolog 3 promotes colorectal cancer progression through mutual regulation of histone deacetylase 7

**DOI:** 10.1002/mco2.576

**Published:** 2024-05-31

**Authors:** Pengyan Lu, Shumin Deng, Jiaxin Liu, Qing Xiao, Zhengwei Zhou, Shuojie Li, Jiaxuan Xin, Guang Shu, Bo Yi, Gang Yin

**Affiliations:** ^1^ Department of Pathology Xiangya Hospital, School of Basic Medical Sciences, Central South University Changsha China; ^2^ Department of Pathology The Third Xiangya Hospital, Central South University Changsha China; ^3^ Department of Gastrointestinal Surgery The Third Xiangya Hospital, Central South University Changsha China; ^4^ National Clinical Research Center for Geriatric Disorders, Xiangya Hospital, Central South University Changsha China; ^5^ China‐Africa Research Center of Infectious Diseases, School of Basic Medical Sciences, Central South University Changsha Hunan Province China

**Keywords:** ceRNA, colorectal cancer, HDAC7, migration, TTYH3

## Abstract

Colorectal cancer (CRC) is one of the leading cancers worldwide, with metastasis being a major cause of high mortality rates among patients. In this study, dysregulated gene Tweety homolog 3 (TTYH3) was identified by Gene Expression Omnibus database. Public databases were used to predict potential competing endogenous RNAs (ceRNAs) for TTYH3. Quantitative real‐time polymerase chain reaction, western blot, and immunohistochemistry were utilized to analyze TTYH3 and histone deacetylase 7 (HDAC7) levels. Luciferase assays confirmed miR‐1271‐5p directly targeting the 3′ untranslated regions of TTYH3 and HDAC7. In vitro experiments such as transwell and human umbilical vein endothelial cell tube formation, as well as in vivo mouse models, were conducted to assess the biological functions of TTYH3 and HDAC7. We discovered that upregulation of TTYH3 in CRC promotes cell migration by affecting the Epithelial–mesenchymal transition pathway, which was independent of its ion channel activity. Mechanistically, TTYH3 and HDAC7 functioned as ceRNAs, reciprocally regulating each other's expression. TTYH3 competes for binding miR‐1271‐5p, increasing HDAC7 expression, facilitating CRC metastasis and angiogenesis. This study reveals the critical role of TTYH3 in promoting CRC metastasis through ceRNA crosstalk, offering new insights into potential therapeutic targets for clinical intervention.

## Graphical abstract

Schematic description of the main molecular mechanism involved in the critical role of TTYH3 in the regulation of CRC migration and angiogenesis via ceRNA mechanism. TTYH3 facilitates the advancement of CRC via interacting with HDAC7 through ceRNA crosstalk, namely by binding miR‐1271‐5p, rather than relying on its chloride ion channels activity.

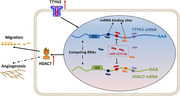

## 1 INTRODUCTION

Colorectal cancer (CRC) is a prevalent malignancy of the digestive system, ranking third in incidence rate at 10.0% and second in mortality rate at 9.4%^1^ among all cancers. The increasing number of CRC patients worldwide poses a severe threat to their survival.^2^ Despite significant advances in CRC diagnosis and treatment, patients with distant metastasis exhibit high mortality and dismal prognosis.^3^ The underlying mechanisms of CRC metastasis are still incompletely elucidated.

TTYH3 is the third member of the Tweety homologs (TTYHs) in mammals, which consists of TTYH1, TTYH2, and TTYH3. These highly conserved members encode gated chloride ion channels that participate in various cellular processes, such as cell volume and calcium activity regulation, cell adhesion, cell division, as well as tumorigenesis.^4^, ^5^ TTYH1 is involved in the maintenance of neural stem cell status, cell proliferation, and filopodia formation during neuronal development, while TTYH2 and TTYH3 potentially play roles in tissue formation, embryonic development, and immune response to pathogen‐associated molecules.^6^, ^7^, ^8^ TTYH1 encodes a volume‐sensitive chloride channel protein,^9^ while TTYH3 encodes a large‐conductance chloride channel activated by Ca^2+^ ^10^. TTYH3 is primarily expressed in excitable tissues, including the brain, heart, and skeletal muscle.^10^ In recent years, studies have demonstrated the involvement of TTYHs in tumorigenesis. For instance, upregulation of TTYH2 has been identified in renal cell and colon carcinoma.^11^, ^12^ Studies have highlighted its role in promoting colon cancer cell proliferation^12^ as well as osteosarcoma cell migration and invasion.^13^ Recent studies have reported the role of the TTYH3/MK5 axis in regulating GSK‐3β/β‐catenin signaling in hepatocellular carcinoma,^14^ and its involvement in bladder cancer progression through the FGFR1/H‐Ras/A‐Raf/MEK/ERK pathway.^15^ Nevertheless, the function and underlying mechanism of TTYH3 in CRC remain unclear. Our study demonstrates that TTYH3 is associated with poor prognosis of CRC patients and plays vital roles in promoting CRC migration independent of its chloride channel activity.

Different RNA transcripts compete for a limited pool of miRNAs through shared micro RNAs (miRNAs) response elements (MREs), effectively leading to the formation of competitive endogenous RNAs (ceRNAs). This intricate ceRNA network enables mutual regulation of these transcripts in expression levels.^16^ It has been demonstrated in various species that pseudogenes, mRNAs, lncRNAs, and circular RNAs (circRNAs), which can function as ceRNAs,^16^, ^17^ are widely involved in carcinogenesis.^18^ Extensive research has been conducted on non‐coding RNA‐related ceRNA mechanisms, but there is currently limited studies on the ceRNA mechanism between coding RNAs. Drawing from a study by Tay et al. in *Cell*,^19^, ^20^ which proposed ceRNA mechanism involving PTEN competing with other coding transcripts by competitively binding endogenous “miRNA pool,” achieving cross‐regulation in expression levels. Thus, we speculate that TTYH3, encoding a chloride ion channel, may regulate other coding genes through ceRNA crosstalk, thereby exerting its biological function in promoting CRC progression.

Current understanding of the regulatory mechanism of TTYH3 in CRC remains limited. Here, we revealed the role of TTYH3 in regulating CRC progression via ceRNA crosstalk, focusing on the interplay between two protein‐coding transcripts, TTYH3 and histone deacetylase 7 (HDAC7). We discovered that TTYH3 exerts its function via reciprocal ceRNA interaction, rather than through ion channel characteristics. Further study demonstrated that TTYH3 competes with HDAC7 for shared miR‐1271‐5p to facilitate CRC metastasis.

## 2 RESULTS

### 2.1 TTYH3 is highly expressed in CRC and is associated with poor prognosis

**FIGURE 1:**
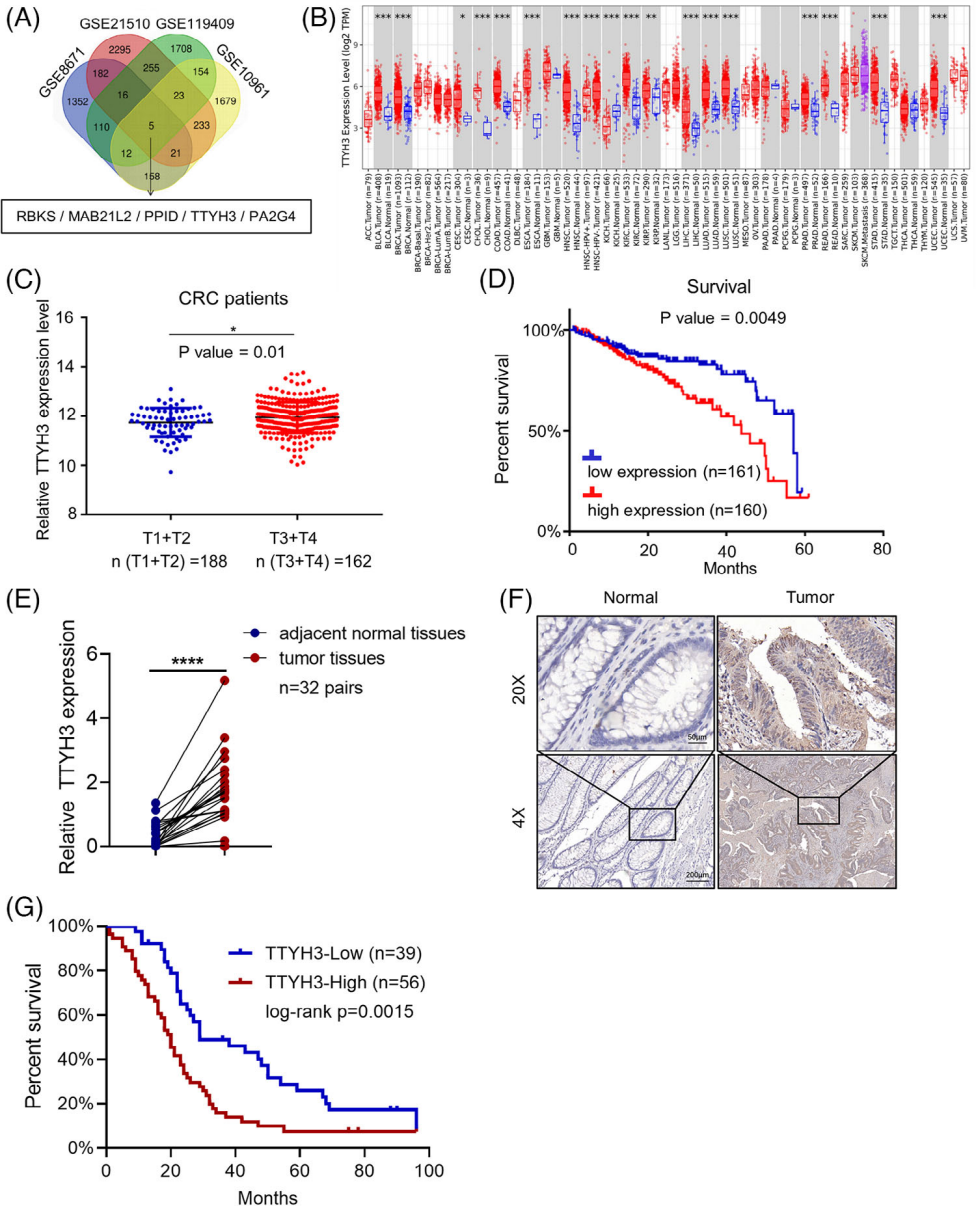
Elevated TTYH3 expression was correlated with unfavorable prognosis. (A) Venn diagram showed the intersection of GSE8671, GSE21510, GSE119409, and GSE10961. (B) Expression of TTYH3 in tumor and normal tissues. Red boxplot: tumor tissues; Blue boxplot: normal tissues; purple boxplot: metastatic tissues. The data was obtained from the TIMER database. (C and D) TTYH3 expression in different tumor stages of CRC patients (C); relationship between TTYH3 levels and the overall survival of CRC patients by Kaplan–Meier analysis (D). Data was gained from the LinkedOmics datasets. (E) Relative TTYH3 expression in clinical tissues by qPCR. (F) Representative images of TTYH3 protein levels in clinical tissue samples. Top: magnification, ×200. Scale bar: 50 μm; Bottom: magnification, ×40. Scale bar: 200 μm. (G) The relationship between TTYH3 levels and CRC patients overall survival by Kaplan–Meier analysis. All experiments were independently repeated three times. *p < 0.05, ****p < 0.0001. CRC, colorectal cancer; qPCR, quantitative real‐time polymerase chain reaction; IHC, immunohistochemistry.

We utilized the Gene Expression Omnibus (GEO) database, including GSE8671, GSE21510, GSE119409, and GSE10961, to detect genes differentially expressed in CRC. Microarray results identified five potential genes with abnormal expression in CRC (Figure 1A). Further analysis using The Cancer Genome Atlas (TCGA) database showed that RBKS and MAB21L2 were downregulated (Figure S1A and B) while TTYH3, PA2G4, and PPID were upregulated (Figure 1B and S1C, D) in CRC. Subsequently, we analyzed the clinical stages of CRC patients in the Linkedomics database, TTYH3 was chosen as our research focus based on its remarkably higher expression in advanced‐stage tumor tissues (Figure 1C and S1E, F). Moreover, patients with elevated TTYH3 levels had considerably poorer 5‐year survival rates (Figure 1D).

To further investigate TTYH3 expression in CRC, we collected and examined TTYH3 expression in 32 paired fresh CRC and adjacent normal tissues. Results showed that TTYH3 was highly expressed in CRC when compared with adjacent normal tissues (Figure 1E). Additionally, we explored the impact of TTYH3 on CRC progression by evaluating clinical paraffin‐embedded tissue samples from 95 CRC patients and 52 matched adjacent normal colon tissues. The results demonstrated that TTYH3 expression was higher in tumor than in normal tissues (Figure 1F and S1G). Moreover, the correlation between TTYH3 protein expression and clinicopathological characteristics among CRC patients was evaluated, which revealed that elevated TTYH3 levels were significantly linked to poor histological differentiation, advanced TNM stage, and distant metastasis (Table 1). Next, we performed a Kaplan–Meier survival analysis based on the TTYH3 expression levels in 95 CRC patients. The results suggested that CRC patients with high TTYH3 levels exhibited a shorter overall survival rates than those with relatively low levels (Log‐Rank, *p* < 0.05) (Figure 1G).

**TABLE 1:** The association between TTYH3 levels with clinicopathological characteristics of CRC patients.

	Expression of TTYH3		
Variable	Low (*n* = 39)	High (*n* = 56)	* *p* Value
Ages (years)			0.4604
≥60	19	23	
<60	20	33	
Gender			0.3943
Male	27	34	
Female	12	22	
Histology grade			**0.0272**
Well/moderate	25	23	
Poor	14	33	
TNM stage			**0.0078**
I–II	24	19	
III–IV	15	37	
Distant metastasis during follow‐up			**0.0005**
Yes	13	39	
No	26	17	

The bold number represents the *p* values with significant differences; *χ ^2^ test.

Taken together, these results strongly indicate that TTYH3 might play a critical role in CRC progression.

### 2.2 TTYH3 promotes CRC cell migration independent of its chloride ion channel activity

**FIGURE 2:**
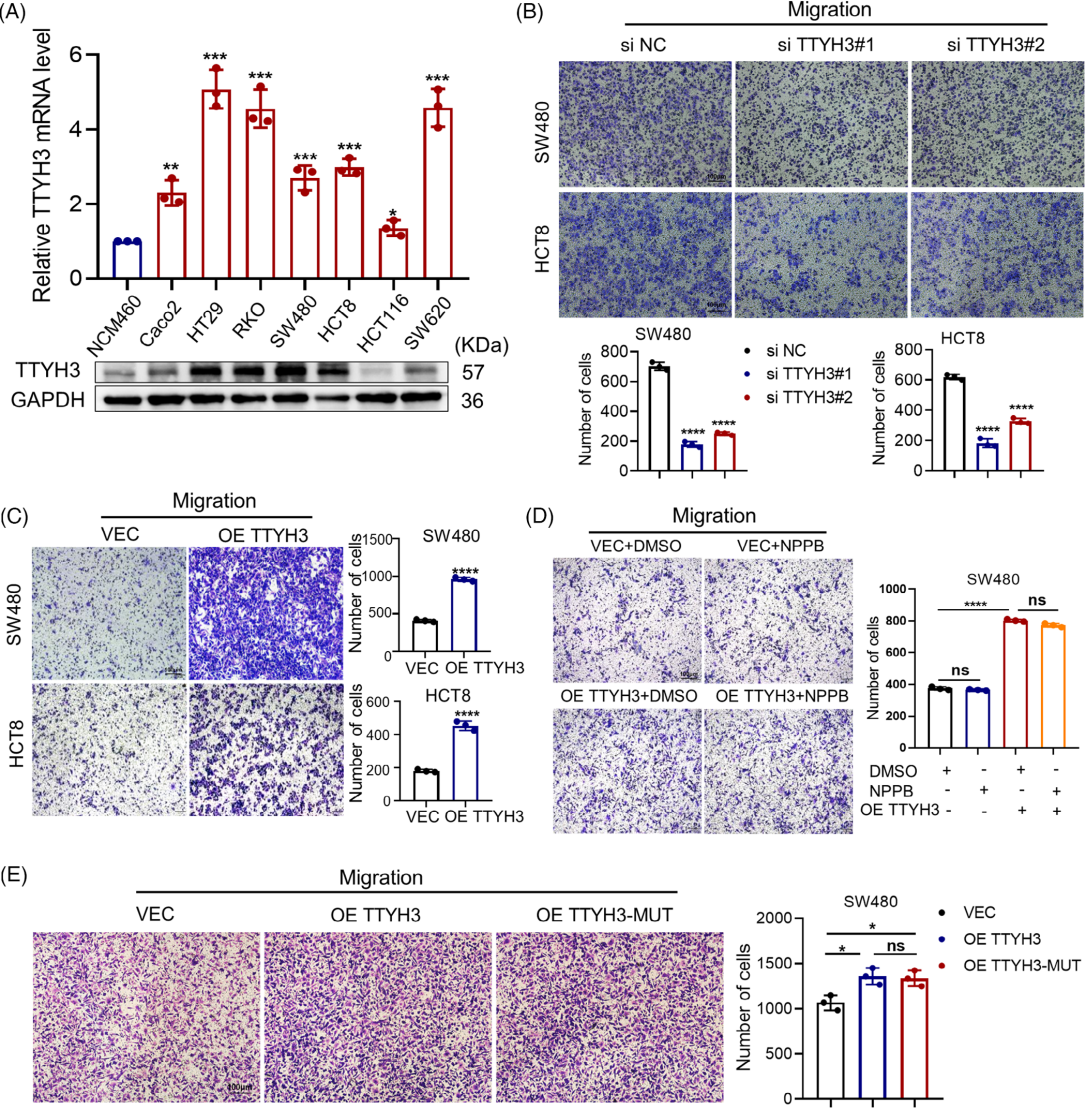
TTYH3 promoted CRC cell migration independently of chloride channel activity. (A) Relative TTYH3 expression in normal colon cell NCM460 and CRC cell lines analyzed by qPCR (top) and western blot (bottom). (B and C) Relative cell motility examined by transwell after TTYH3 knockdown (B) and overexpression (C). (D and E) Transwell assay was utilized to detect the impact on cell motility after NPPB treated (D) or transfected with TTYH3 pore mutant plasmids (E). All experiments were independently repeated three times. *p < 0.05, **p < 0.01, ***p < 0.001, ****p < 0.0001. NPPB, 5‐nitro‐2‐(3‐phenylpropylamino)‐benzoic acid.

To investigate the impact of TTYH3 on the malignant phenotypes of CRC cells, we examined its expression in CRC cell lines (Figure 2A). The results showed that TTYH3 was generally highly expressed in CRC cells. Considering the varying migratory abilities among different cell lines, we selected SW480 and HCT8 with moderate TTYH3 levels for subsequent experiments. Then, TTYH3 was knocked down and overexpressed in these two cell lines, respectively (Figure S2A). The results revealed that knocking down TTYH3 markedly inhibited CRC cell migration (Figure 2B), as well as the invasive capability of SW480 cells (Figure S2B). Besides, we also performed a wound healing assay on HT29 cells, which exhibited the highest TTYH3 expression levels. The results were consistent with those observed in SW480 and HCT8 cell lines. (Figure S2C). Conversely, TTYH3 overexpression notably promoted SW480 and HCT8 cells migration (Figure 2C) and enhanced the invasion of SW480 cells (Figure S2D). Given that TTYH3 encodes a chloride ion channel, multiple studies have highlighted the significant role of chloride ion channels in cancer progression.^21^ We further explored whether TTYH3 exerts its effects through chloride ion channels. To achieve this, we treated CRC cells with the specific Ca^2+^‐dependent chloride ion channel inhibitor 5‐nitro‐2‐(3‐phenylpropylamino)‐benzoic acid (NPPB). Surprisingly, NPPB treatment had no significant effect on CRC cell migration, while overexpressing TTYH3 still greatly promoted SW480 cell migration (Figure 2D). In addition, according to the paper mentioned,^10^ we constructed a plasmid with mutations in the pore region of the ion channel for further examination. The results demonstrated that mutation did not affect the promotion effect of TTYH3 on CRC cell migration (Figure 2E), which was consistent with results previously obtained from inhibitor treatment.

These findings suggest that TTYH3 can facilitate CRC cell migration in vitro, independent of the properties associated with chloride ion channels.

### 2.3 HDAC7 acting as a reciprocal ceRNA of TTYH3 was upregulated in CRC

**FIGURE 3:**
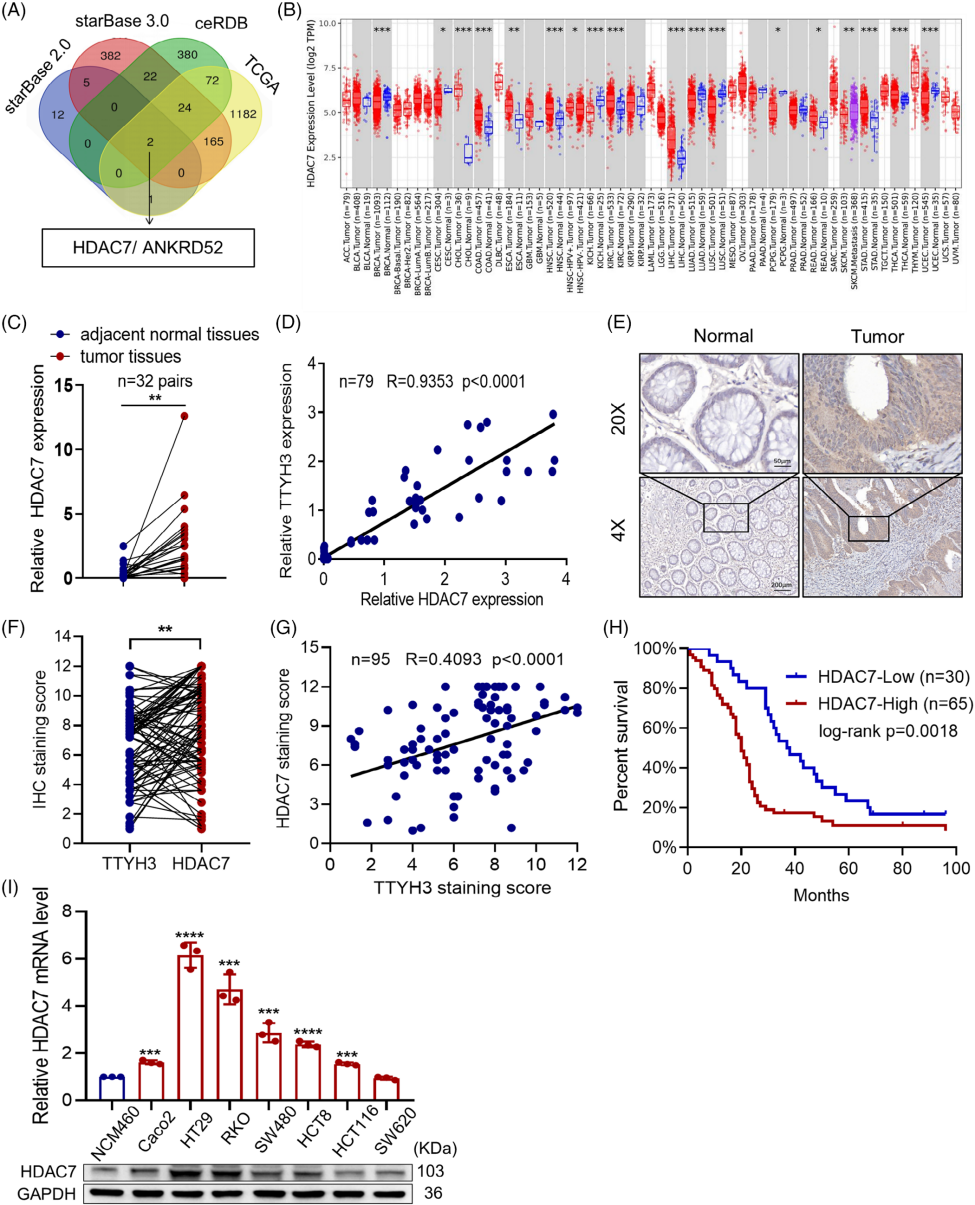
HDAC7 functioning as a ceRNA of TTYH3 was augmented in CRC. (A) Venn diagram displayed the intersection of TTYH3 candidate ceRNA molecules. (B) Expression of HDAC7 in tumor and normal tissues. Red boxplot: tumor tissues; blue boxplot: normal tissues; Purple boxplot: metastatic tissues. Data were obtained from the TIMER database. (C) Relative HDAC7 expression in clinical tissues by qPCR. (D) The correlation in mRNA expression between TTYH3 and HDAC7 in 79 CRC tissues by qPCR. (E) Representative images showed relative HDAC7 protein levels in clinical tissue samples by IHC. Top: magnification, ×200. Scale bar: 50 μm; Bottom: magnification, ×40. Scale bar: 200 μm. (F) IHC staining quantification of TTYH3 and HDAC7 expression in 95 CRC tissues. (G) The correlation in protein expression between TTYH3 and HDAC7 in 95 CRC tissues by IHC. (H) Kaplan–Meier analysis of the overall survival in CRC patients with low or high HDAC7 levels. (I) Relative HDAC7 expression in normal colon cell NCM460 and CRC cell lines analyzed by qPCR (top) and western blot (bottom). All experiments were independently repeated three times. *p < 0.05, **p < 0.01, ***p < 0.001, ****p < 0.0001. ceRNA, competitive endogenous RNA.

To elucidate the underlying mechanism behind TTYH3‐mediated promotion of cell migration, we proposed the possibility of TTYH3 upregulation influencing the expression of other oncogenes, thereby facilitating CRC malignancy. Previous papers have reported on the ceRNA mechanism^19^ which may shed light on this aspect. Hence, We used the Starbase 3.0 and TCGA databases (to identify genes positively associated with TTYH3 in CRC) to predict potential ceRNA candidates of TTYH3. The intersection of these databases revealed two genes, HDAC7 and ANKRD52 (Figure 3A). Next, the TCGA database analysis showed that HDAC7 was highly expressed (Figure 3B and S3A), whereas ANKRD52 was downregulated in CRC (Figure S1H and S3B). Additionally, GEPIA2 database further revealed a stronger positive correlation between TTYH3 and HDAC7 compared with ANKRD52. (Figure S3C and D). In light of all the above, we chose HDAC7 instead of ANKRD52 for further investigation. Before identifying HDAC7 as the possible ceRNA candidate for TTYH3, we first investigated the potential interaction between TTYH3 and HDAC7. To explore this, we employed the co‐immunoprecipitation (co‐IP) assay on cells transfected with plasmids overexpressing TTYH3 (fused with GFP tag) and HDAC7 (fused with Flag tag). Results indicated no direct protein‐protein interaction between TTYH3 and HDAC7 (Figure S3E). Besides, no significant changes were observed in TTYH3 protein levels when treated SW480 cells with either CHX alone or in combination with MG132 (Figure S3F). Thus, we tentatively ruled out the post‐translational ubiquitination modification of TTYH3 by HDAC7. Considering this, we speculate that there might be a ceRNA regulatory interaction between TTYH3 and HDAC7. Additionally, database analysis showed that HDAC7 expression was substantially higher in distant metastatic tumor tissues than in primary CRC tissues (Figure S3G). Higher tumor stages were associated with elevated HDAC7 expression levels as well (Figure S3H). Furthermore, quantitative real‐time polymerase chain reaction (qRT‐PCR) analysis on 32 pairs of fresh CRC and adjacent normal tissues confirmed elevated levels of HDAC7 in cancer tissues (Figure 3C). To validate the correlation between TTYH3 and HDAC7 in CRC, we examined their levels in 79 CRC tissues. The results revealed a strong positive correlation between them (Pearson correlation coefficient of 0.9353) (Figure 3D), consistent with GEPIA2 database. Similarly, we assessed the correlation between HDAC7 expression and clinicopathological features in 95 CRC patients (Table 2). The findings showed that HDAC7 levels were positively correlated with histological differentiation, clinical stage and distant metastasis during follow‐up. Immunohistochemistry (IHC) analysis also revealed markedly elevated levels of HDAC7 in CRC tissues compared with normal tissues (Figure 3E and S1I). Furthermore, IHC experiments were also conducted to investigate the expression pattern of TTYH3 and HDAC7 in the same tissue samples (Figure S3I). Results demonstrated that both TTYH3 and HDAC7 exhibited high expression levels in one tissue (top) and low expression levels in another (bottom). Quantitative IHC analysis in 95 clinical CRC patient samples also illustrated that tissues with high TTYH3 levels exhibited elevated levels of HDAC7, whereas tissues with low TTYH3 expression displayed decreased HDAC7 levels (Figure 3F). And the correlation analysis demonstrated their positive correlation (*R* = 0.4093) in CRC tissues as well (Figure 3G). Further, Kaplan–Meier survival analysis of these 95 samples was performed based on HDAC7 levels, which indicated that CRC patients with higher HDAC7 levels exhibited a lower overall survival rate (Figure 3H). We subsequently examined HDAC7 expression in CRC cell lines (Figure 3I) and selected SW480 and HCT8, which demonstrate moderate HDAC7 levels, for further investigations. The results above indicate a potential role of HDAC7 as a ceRNA for TTYH3, demonstrating its upregulation in CRC. Additionally, it is suggested that TTYH3 and HDAC7 are co‐expressed in clinical CRC tissues.

**TABLE 2:** The relationship of HDAC7 expression in clinicopathological characteristics of CRC patients.

	Expression of HDAC7		
Variable	Low (*n* = 30)	High (*n* = 65)	* *p* Value
Ages (years)			0.0968
≥60	17	25	
<60	13	40	
Gender			0.7344
Male	20	41	
Female	10	24	
Histology grade			0.0325
Well/moderate	20	28	
Poor	10	37	
TNM stage			**0.0162**
I–II	19	24	
III–IV	11	41	
Distant metastasis during follow‐up			**0.0162**
Yes	11	41	
No	19	24	

The bold number represents the *p* values with significant differences; *χ ^2^ test.

### 2.4 TTYH3 and HDAC7 co‐express and mutually regulate their expression levels in CRC

**FIGURE 4:**
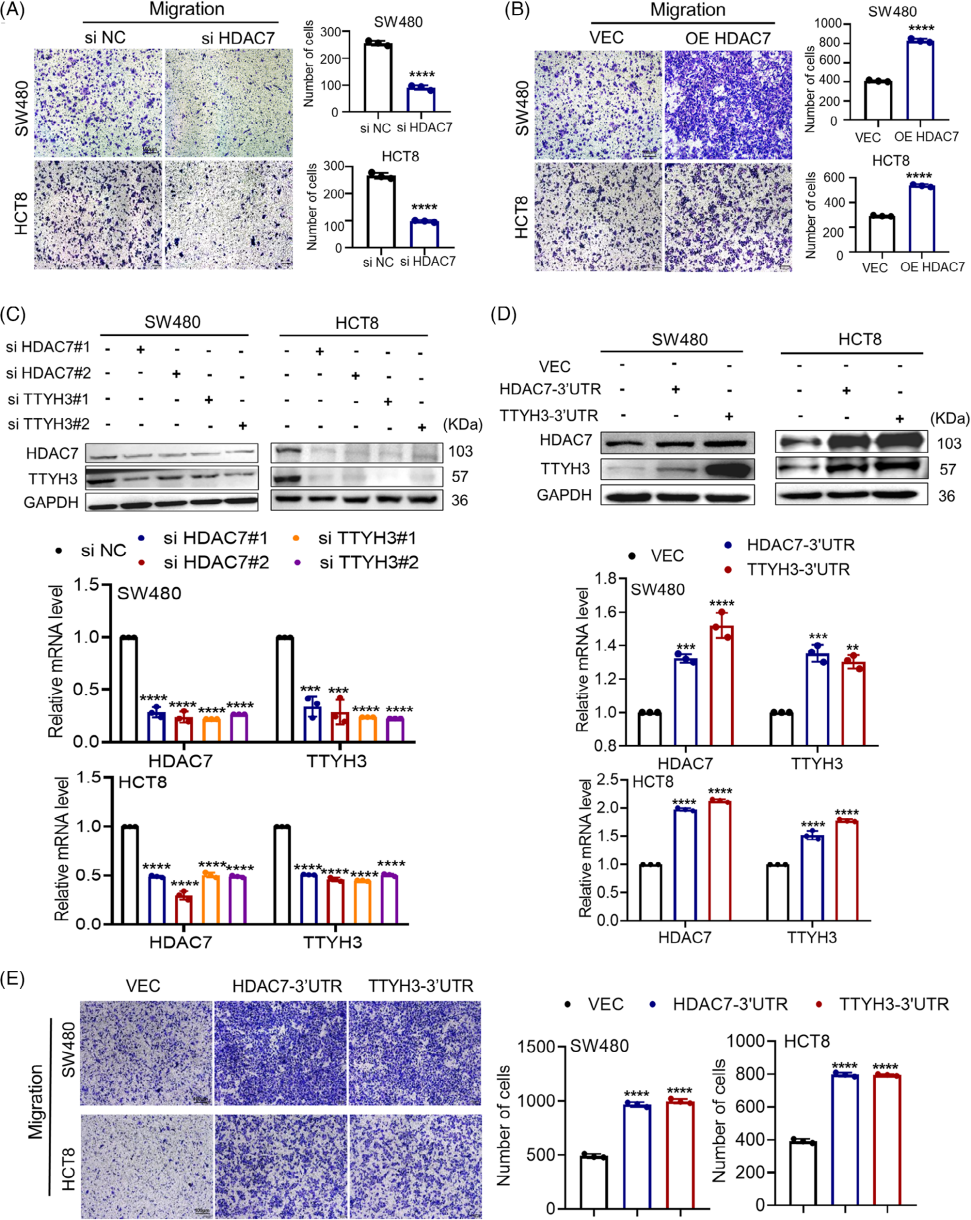
TTYH3 and HDAC7 co‐express and reciprocally regulate each other in CRC. (A and B) Relative cell motility examined by transwell after HDAC7 knockdown (A) and overexpression (B). (C and D) Relative TTYH3 and HDAC7 expression detected by western blot (top) and qPCR (bottom) in SW480 and HCT8cells after knockdown (C) and overexpression (D). (E) Relative cell motility examined by transwell after overexpressing 3′UTRs respectively. All experiments were independently repeated three times. **p < 0.01, ***p < 0.001, ****p < 0.0001. 3′UTR, 3′ untranslated region.

It was reported that HDAC7 promotes tumor growth and invasion in CRC.^22^ Moreover, we aimed to use loss and gain‐of‐function experiments to investigate HDAC7's role in CRC. We knocked down and overexpressed HDAC7 in SW480 and HCT8 cells (Figure S2E). We found that HDAC7 knockdown suppressed in vitro migration of CRC cells (Figure 4A), while overexpression facilitated it (Figure 4B). Considering the co‐expression characteristic of ceRNA molecules,^20^ we further explored the correlation between HDAC7 and TTYH3. We unexpectedly discovered that knocking down HDAC7 caused a decrease in both mRNA and protein levels of HDAC7 and TTYH3 (Figure 4C). Similarly, when TTYH3 was knocked down, the expression levels of both HDAC7 and TTYH3 decreased as well (Figure 4C). Conversely, overexpression of the 3′ untranslated regions (3′UTR) of HDAC7 increased both HDAC7 and TTYH3 mRNA and protein levels; transfection of TTYH3‐3′UTR yielded similar results as well (Figure 4D). These results indicate that TTYH3 and HDAC7 are co‐expressed in CRC cell lines. Moreover, overexpression of the 3′UTR of either TTYH3 or HDAC7 significantly enhanced cell migration (Figure 4E).

The data above demonstrates that TTYH3 and HDAC7 co‐regulate CRC cell migration, with both the 3′UTR and coding sequence (CDS) regions playing crucial roles in promoting this process. These results suggest a ceRNA relationship between HDAC7 and TTYH3, which contributes to CRC cell migration.

### 2.5 TTYH3 and HDAC7 competitively bind miR‐1271‐5p in CRC

**FIGURE 5:**
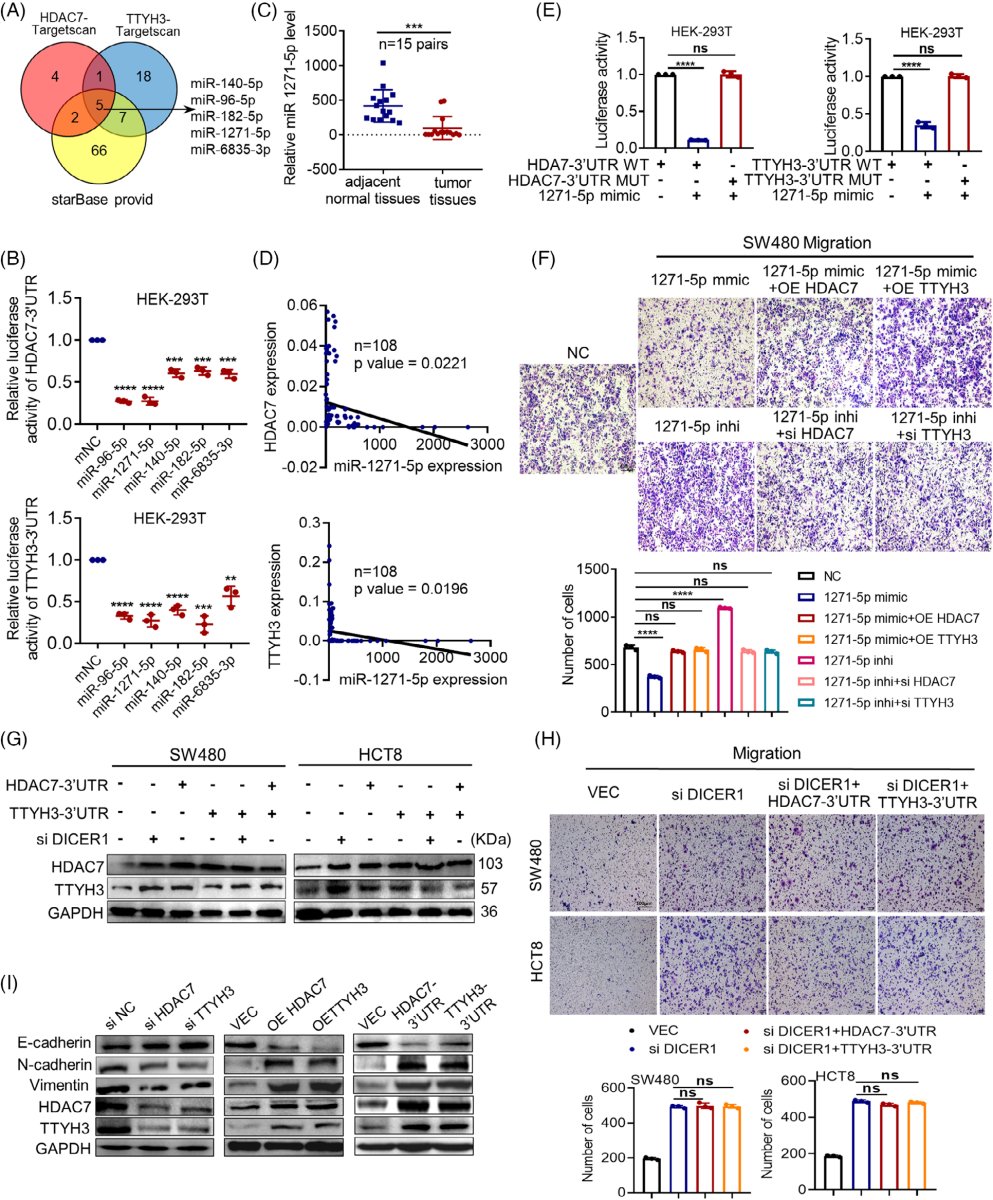
TTYH3 and HDAC7 compete for binding miR‐1271‐5p in CRC. (A) Prediction of miRNAs that target both TTYH3 and HDAC7. (B) Detection of the luciferase activity in HEK‐293T after transfecting different miRNA mimics together with HDAC7‐3′UTR (top) or TTYH3‐3′UTR (bottom). (C) Relative miR‐1271‐5p expression in clinical tissues by qPCR. (D) Analysis of the correlation between miR‐1271‐5p and HDAC7 (top) or TTYH3 (bottom) in 108 CRC tissues by qPCR. (E) Relative luciferase activities in HEK‐293T cotransfected with miR‐1271‐5p mimic and HDAC7‐3′UTR (left) or TTYH3‐3′UTR (right) WT/MUT plasmid. (F) Relative cell motility of rescue assay detected by transwell. (G) Relative HDAC7 and TTYH3 expression after DICER1 knocked down by western blot. (H) Relative cell motility after DICER1 knocked down by transwell. (I) Relative expression of TTYH3, HDAC7 and EMT‐related molecules detected by western blot. All experiments were independently repeated three times. **p < 0.01, ***p < 0.001, ****p < 0.0001. EMT, epithelial–mesenchymal transition; HEK, human embryonic kidney; WT, wild‐type.

Protein‐coding mRNA transcripts can engage in ceRNA crosstalk by sequestering shared miRNAs, thereby endowing the mRNA transcripts with previously unrecognized non‐coding functions that are encrypted within the mRNA itself. This crosstalk among mRNA transcripts occurs through a “language” based on MREs.^20^ Therefore, to identify potential miRNAs involved, we utilized Targetscan to predict miRNAs targeting HDAC7 or TTYH3 and Starbase3.0 to find miRNAs that targeting both TTYH3 and HDAC7. The results revealed five miRNAs (Figure 5A). Further investigation by luciferase reporter assays demonstrated that miR‐96‐5p and miR‐1271‐5p significantly reduced the luciferase activity of both HDAC7 and TTYH3 3′UTRs (Figure 5B). Also, transfecting SW480 cells with these two miRNA mimics reduced both TTYH3 and HDAC7 protein levels (Figure S4A). TCGA database showed upregulation of miR‐96‐5p (Figure S4B) and downregulation of miR‐1271‐5p (Figure S4C) in CRC when compared with normal tissues. These findings were further validated through qPCR analysis of 15 paired fresh CRC and adjacent normal tissues (Figure 5C and S4D). Given that TTYH3 and HDAC7 were upregulated in CRC, it is reasonable to speculate that the miRNAs targeting them in CRC may be downregulated. Therefore, miR‐1271‐5p was selected for further investigation. miR‐1271‐5p expression was negatively correlated with both TTYH3 and HDAC7 in 108 CRC tissues by qRT‐PCR assay (Figure 5D). Besides, inhibiting miR‐1271‐5p led to an evident increase in HDAC7 protein levels, further supporting the negative regulatory relationship between HDAC7 and miR‐1271‐5p (Figure S4E). Moreover, Targetscan was used to predict direct binding sites for miR‐1271‐5p on both 3′UTRs of HDAC7 and TTYH3, and mutant plasmids were constructed based on the binding sites for luciferase reporter assays (Figure S4F). The results indicated that the mutation in 3′UTR binding sites of TTYH3 or HDAC7 notably augmented the luciferase activity (Figure 5E), further confirming the direct targeting of miR‐1271‐5p at the 3′UTRs of TTYH3 and HDAC7. On top of that, we assessed the impact of miR‐1271‐5p on cell migration (Figure 5F), which ascertained that miR‐1271‐5p mimics visibly suppressed CRC migration. Moreover, the inhibitory effect mediated by miR‐1271‐5p was restored upon the overexpression of TTYH3 or HDAC7 (Figure 5F). Conversely, miR‐1271‐5p inhibitor promoted migration, and this promoting effect was rescued by either HDAC7 or TTYH3 knockdown. These results conclusively revealed that miR‐1271‐5p inhibits cell migration by targeting both TTYH3 and HDAC7.

To further validate the ceRNA association between TTYH3 and HDAC7, we focused on DICER, a key RNase III member in miRNAs maturation.^23^ In SW480 cells, either knocking down DICER1 alone or overexpressing TTYH3‐3′UTR or HDAC7‐3′UTR individually augmented both TTYH3 and HDAC7 protein levels (Figure 5G, left). Interestingly, when DICER1 knockdown was combined with the overexpression of either TTYH3‐3′UTR or HDAC7‐3′UTR, no significant alteration in protein levels was observed compared with the group of knocking down DICER1 alone (Figure 5G, left). Similar results were identified in HCT8 cells (Figure 5G, right). Furthermore, knocking down DICER1 notably promoted cell migration(Figure 5H). However, when overexpressing TTYH3‐3′UTR or HDAC7‐3′UTR respectively together with DICER1 knockdown simultaneously, the migration‐promoting effect demonstrated no significant difference compared with DICER1 knockdown alone (Figure 5H). These findings suggest that TTYH3 and HDAC7 compete for binding miR‐1271‐5p in CRC cells, and their mutual regulation disrupted upon DICER1 knockdown.

Additionally, HDAC7 was reported to affect epithelial–mesenchymal transition (EMT) pathway in prostate cancer.^24^ Hence, we examined TTYH3 and HDAC7 in CRC cells to study their impact on EMT‐related molecules (Figure 5I and S4G, H). The data revealed that both TTYH3 and HDAC7, as well as their 3′UTRs, upregulated Vimentin and N‐cadherin and downregulated E‐cadherin both in mRNA and protein levels. The findings indicate that TTYH3 and HDAC7 affect EMT‐related pathway, thereby influencing CRC cell migration.

### 2.6 TTYH3 compete with HDAC7 for binding miR‐1271‐5p, promoting human umbilical vein endothelial cell tube formation in vitro

**FIGURE 6:**
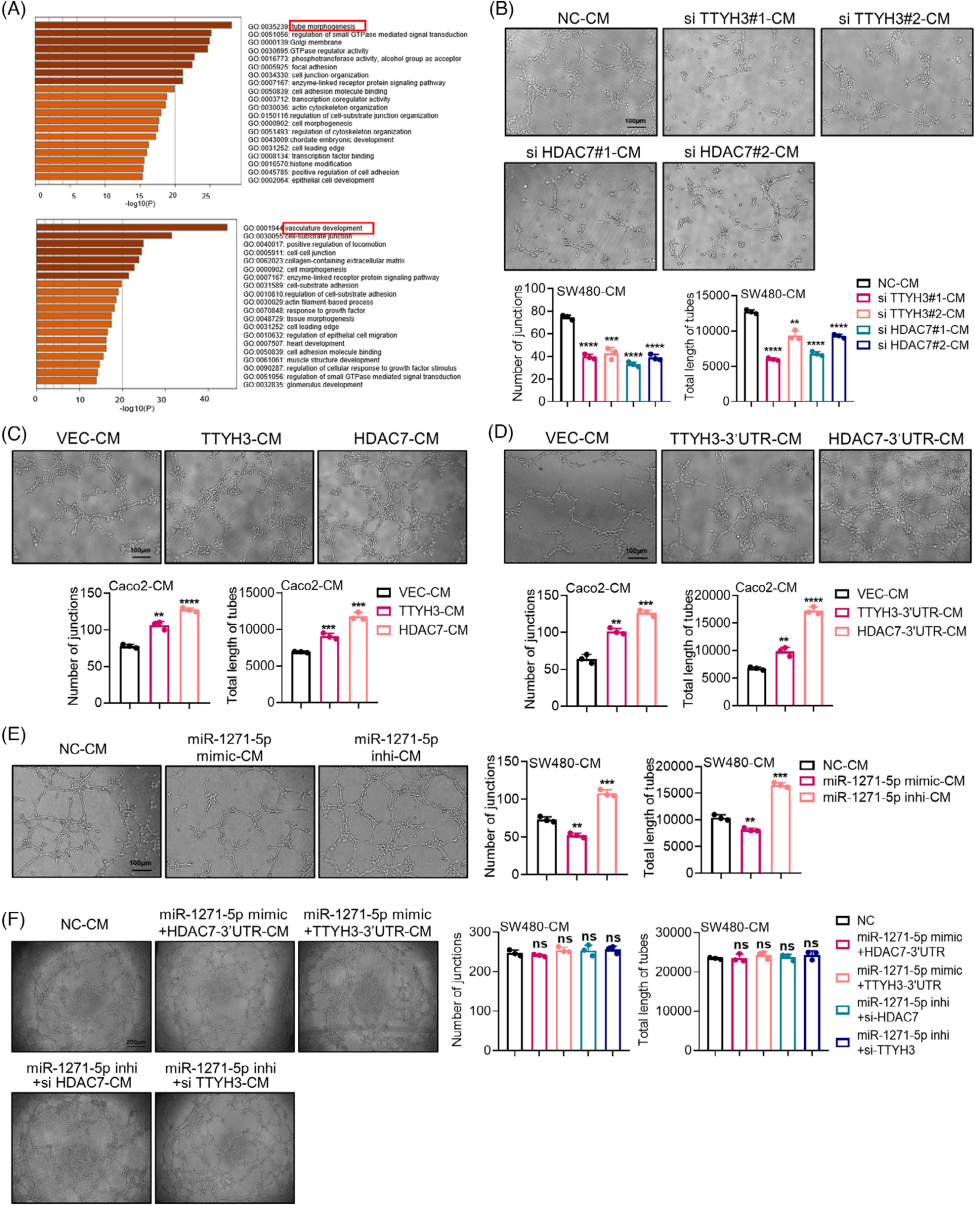
TTYH3 promoted tube formation of HUVECs in vitro by competing for miR‐1271‐5p with HDAC7. (A) Go analysis of TTYH3 (top) and HDAC7 (bottom) by Metascape database. (B and C) Images of HUVEC tube formation after downregulation (B) or upregulation (C) of TTYH3 or HDAC7. (D) Images of HUVEC tube formation after upregulation of TTYH3‐3′UTR or HDAC7‐3′UTR. (E) Examination of miR‐1271‐5p impact on tube formation. (F) Detection of rescue assay on tube formation. Number of tube junctions and total length demonstrate the angiogenesis ability in each group. Magnification, ×40. Scale bars, 200 μm; Magnification, ×100. Scale bars, 100 μm. All experiments were independently repeated three times. **p < 0.01, ***p < 0.001, ****p < 0.0001. GO, Gene Ontology; HUVEC, human umbilical vein endothelial cell.

Tumor metastasis is closely associated with tumor angiogenesis, and HDAC7 plays vital roles in vascular repair, regeneration, and stability maintenance. Previous studies have highlighted HDAC7's involvement in vascular development in glioblastoma^25^ and chronic obstructive pulmonary disease.^26^ Given the ceRNA interaction between TTYH3 and HDAC7, we hypothesized that TTYH3 might also contribute to promoting angiogenesis. To investigate this, we screened for TTYH3 or HDAC7 correlated genes in the TCGA database and conducted a Gene Ontology (GO) enrichment analysis, which revealed that genes positively associated with TTYH3 (Figure 6A, top) or HDAC7 (Figure 6A, bottom) were enriched in angiogenesis‐related pathways. Subsequently, experimental assays were carried out to examine their effect on angiogenesis. Initially, we knocked down TTYH3 or HDAC7 in SW480 cells and overexpressed both their CDS or 3′UTRs respectively in Caco2 cells (Figure S2F). Then, conditioned medium (CM) from CRC cells was collected to culture human umbilical vein endothelial cell (HUVEC) for tube formation assays. The results revealed that TTYH3 or HDAC7 knockdown inhibited HUVEC tube formation (Figure 6B), while overexpression of TTYH3 or HDAC7 promoted it (Figure 6C). Besides, overexpressing TTYH3‐3′UTR or HDAC7‐3′UTR fostered HUVEC tube formation as well (Figure 6D). Furthermore, overexpressing TTYH3‐3′UTR or HDAC7‐3′UTR enhanced both the proliferation and migration of HUVECs. (Figure S4I and J), which was crucial for HUVEC tube formation in vitro. Thereafter, we explored the effect of miR‐1271‐5p, which targets both TTYH3 and HDAC7, on HUVEC tube formation. Results revealed that the miR‐1271‐5p mimic repressed tube formation, while the inhibitor accelerated it (Figure 6E). Additionally, the inhibitory effect of TTYH3 or HDAC7 knockdown on HUVEC tube formation could be restored by the miR‐1271‐5p inhibitor (Figure 6F). Similarly, the promoting effect of TTYH3‐3′UTR or HDAC7‐3′UTR overexpression on HUVEC tube formation could be reinstated by miR‐1271‐5p mimic transfection (Figure 6F). Taken together, our findings suggest that TTYH3 promotes HUVEC tube formation by competing with HDAC7 for binding miR‐1271‐5p.

### 2.7 The transcript of TTYH3 acts as ceRNA for HDAC7, promoting both CRC metastasis and angiogenesis in vivo

**FIGURE 7:**
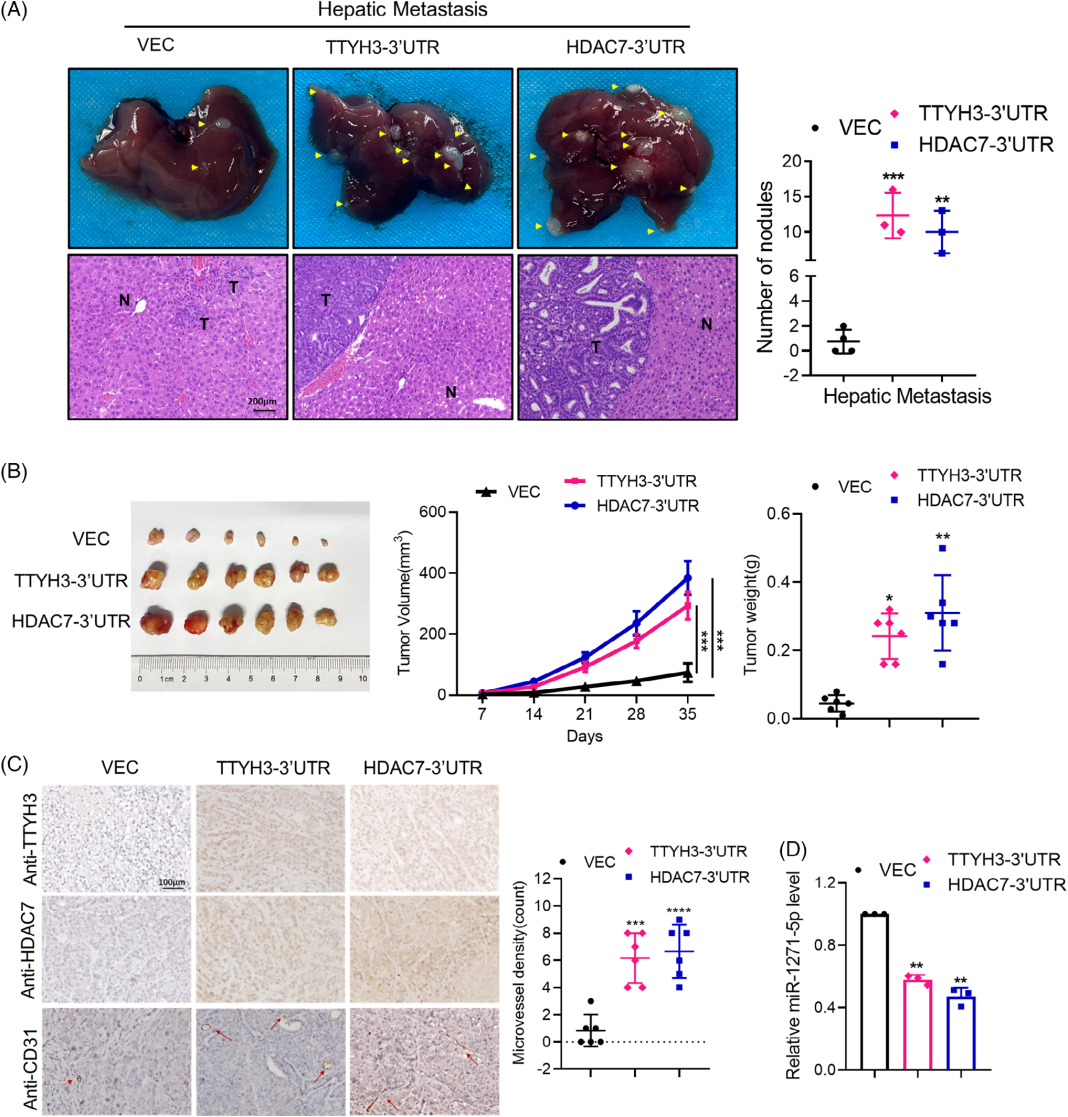
TTYH3 transcript acts as ceRNA for HDAC7, facilitating both CRC metastasis and angiogenesis in vivo. (A) Representative images of hepatic metastatic lesions in mice models. (B) Representative images of xenografts in nude mice (left) with the measured tumor volume and weight (right) of different groups. (C) Representative IHC images of TTYH3, HDAC7 and CD31 expression in xenografts (left) and the quantification of microvessels in different groups (right). Magnification: 100×. Scale bar: 100 μm. (D) Relative miR‐1271‐5p expression in xenografts of different groups. All experiments were independently repeated three times. *p < 0.05, **p < 0.01, ***p < 0.001, ****p < 0.0001. N, normal, T, tumor.

To ascertain the ceRNA relationship between TTYH3 and HDAC7 in promoting cancer metastasis in vivo, we evaluated the effects of TTYH3‐3′UTR and HDAC7‐3′UTR on tumor cell colonization and dissemination by using nude mice hepatic metastasis models. The results revealed that overexpression of TTYH3‐3′UTR or HDAC7‐3′UTR led to significantly higher hepatic colonization, liver metastatic lesions in comparison with the control group (Figure 7A). These findings suggest that elevated levels of TTYH3‐3′UTR and HDAC7‐3′UTR enhanced tumor colonization and metastasis.

To investigate whether TTYH3 or HDAC7 participate in CRC angiogenesis, we established mice subcutaneous tumor models. We found that mice overexpressing TTYH3‐3′UTR or HDAC7‐3′UTR exhibited significantly larger subcutaneous tumor volumes and weights (Figure 7B). IHC staining also revealed higher microvessel densities in TTYH3‐3′UTR or HDAC7‐3′UTR overexpression groups (Figure 7C). Further assessment of miR‐1271‐5p expression in subcutaneous tumor tissues showed a significant decrease in the overexpression group compared with the control group (Figure 7D). Collectively, these results indicate that TTYH3 and HDAC7 promote CRC metastasis and angiogenesis in vivo via ceRNA regulation.

## 3 DISCUSSION

TTYH3 encodes Ca^2+^‐activated high‐conductivity chloride ion channels.^8^, ^9^ In human beings, TTYH3 expression is primarily observed in excitable tissues. More recently, there has been an increasing number of studies on the role of TTYH3 in tumor progression.^9^ For example, TTYH3 has been shown to facilitate bladder cancer through the FGFR1/H‐Ras/A‐Raf/MEK/ERK pathway,^15^ and its upregulation promotes EMT in cholangiocarcinoma.^27^ But the function of TTYH3 in CRC progression remains unclear. Our study demonstrated the critical role of TTYH3 in promoting CRC metastasis and in regulating EMT‐related molecules expression. Although previous research has suggested that chloride channels are linked to the progression of malignant tumors.^28^ Interestingly, our findings indicate that the TTYH3‐mediated facilitation of CRC cell migration is not reliant on the functionality of chloride ion channels. This is supported by the absence of any observed effects when cells was exposed to a specific channel blocker or treated by the ion channel activity mutant plasmids. However, Wang et al.^14^ demonstrated that TTYH3 increases calcium influx and intracellular chloride concentration in hepatocellular carcinoma, thereby enhancing cellular migration and influencing EMT. This study suggests a potential reliance of TTYH3 on its ion channel activity, which contradicts our findings in CRC. Currently, our research primarily focuses on investigating the role of TTYH3 in CRC. We are uncertain whether the ion channel‐independent characteristics of TTYH3 are specific to CRC or exhibit tumor‐type specificity, as we have yet to explore this issue. Further exploration is essential to shed light on this intriguing subject. In our future research, we aim to delve deeper into unveiling the underlying mechanisms and potential tumor‐specific variations in the functionality of TTYH3. This will help provide a full grasp of TTYH3's role in cancer progression.

ceRNAs have emerged as crucial post‐transcriptional regulators that alters gene expression through miRNA‐mediated manners and are widely engaged in oncogenesis.^17^, ^19^ For instance, LncRNA‐MIAT functions as a ceRNA to sponge miR‐150‐5p, boosting EZH2 expression and facilitating the thyroid cancer development.^29^ PTEN, a key tumor suppressor, and its endogenous protein‐encoded transcripts are known to disrupt PI3K/AKT signaling, exerting growth‐inhibiting and tumor suppressive properties.^20^ Moreover, protein‐coding mRNA transcripts may also engage in ceRNA crosstalk by competitively sequestering common miRNAs, thereby endowing the mRNA transcripts with previously unrecognized non‐coding functions which encrypted within the mRNA. This cross‐talk occurs via a language based on MREs.^20^

In this study, we identified a strong positive correlation between TTYH3 and HDAC7. They are co‐expressed in CRC and can compete for common miRNAs to cross‐regulate their expression levels. Functional experiments indicate their similar biological functions in CRC. The pursuit of HDAC7 inhibitors is currently at the forefront of research and development.^30^ HDAC7 is reported to promote various cancers progression. Direct repression or inactivation of HDAC7 through HDAC1 and HDAC3 inhibition downregulates multiple super‐enhancers (SEs) and SE‐associated oncogenes, suppressing cancer stem cell phenotypes in breast cancer.^31^ HDAC7 has been reported to promote the proliferation, migration, and invasion of nasopharyngeal carcinoma by downregulating miR‐4465 and upregulating EphA2.^32^ Silencing HDAC7 in glioblastoma resets the tumor suppressor activity of STAT3.^25^ Additionally, miR‐489 has been found to target HDAC7, thereby suppressing tumor growth and invasion in CRC.^22^ It is well established that multiple pathways contribute to cancer cell migration.^33^ Our study has revealed that HDAC7 is implicated in regulating EMT‐related molecules like Vimentin, E‐cadherin, and N‐cadherin. Regarding the mechanisms through which EMT is induced by HDAC7 or TTYH3, further investigations have yet been conducted by us. However, Feng et al.^34^ demonstrated the impact of HDAC7 on EMT by upregulating Snail. Specially, Wang et al.^14^ have reported that TTYH3 can impact EMT through GSK3‐β/β‐catenin pathway in hepatocellular carcinoma. Whereas, we focused on elucidating that TTYH3 promotes EMT progression by upregulating HDAC7. Our data do not provide conclusive evidence whether TTYH3 itself can induce EMT in CRC. The involvement of other molecules through which TTYH3 regulates EMT in CRC still unclear. Subsequent experiments will be conducted to delve deeper into this aspect.

HDAC7 plays a crucial role in angiogenesis by promoting endothelial cell tube formation, vascular stability, repair, and regeneration.^35^, ^36^, ^37^, ^38^ Our research revealed that TTYH3 and HDAC7 are co‐expressed in CRC, not only does the CDS but their 3′UTRs also possess biological functions in facilitating HUVEC tube formation and in vivo angiogenesis. Several studies illuminated the intricate mechanisms by which HDAC7 facilitates tube formation. Turtoi et al.^38^ revealed an intriguing epigenetic control of the angiogenesis suppressor gene AKAP12 by HDAC7. They demonstrated that depletion of HDAC7 raised the acetylation of H3 histones associated with the AKAP12 promoter, consequently leading to AKAP12 upregulation. Additionally, Kato et al.^39^ identified an interaction between HDAC7 and hypoxia‐inducible factor 1α (HIF‐1α), further enhancing HIF‐1α transcriptional activity. They observed the co‐translocation of HDAC7 and HIF‐1α to the nucleus under hypoxic conditions, where HDAC7 forms a complex with HIF‐1α and p300, ultimately augmenting the transcriptional activity of HIF‐1α. However, the precise mechanisms by which HDAC7 promotes CRC angiogenesis remain elusive. The comprehensive exploration of the underlying mechanisms by which TTYH3/HDAC7 regulates tube formation remains incomplete. We have yet investigated the specific molecules released by CRC cells that induce HUVEC tube formation.^38^, ^39^ In summary, there is a compelling connection between HDAC7 and HUVEC tube formation. Previous studies have elucidated various molecular mechanisms by which HDAC7 fosters tube formation. Exploring the involvement of HDAC7 in promoting HUVEC tube formation in CRC is an promising avenue. Nevertheless, we are committed to conducting in‐depth investigation of this phenomenon in our future research endeavors.

Numerous long non‐coding RNAs have been identified as regulators of miR‐1271‐5p expression. For instance, lncRNA ZFAS1 suppresses miR‐1271‐5p, leading to HK2 upregulation and glioma progression.^40^ Moreover, FAM201A drives cervical cancer progression by targeting the miR‐1271‐5p/FLOT1 axis, ultimately activating the Wnt/β‐catenin pathway.^41^ Meanwhile, our study uncovered that miR‐1271‐5p was downregulated in CRC, and TTYH3 competes with HDAC7 for sequestering miR‐1271‐5p, promoting angiogenesis and CRC metastasis. Unlike previous findings on chloride channels in tumor progression, we found that TTYH3's migration‐promoting function on CRC cells is separate from its chloride channel activity. Instead, TTYH3 acts as the ceRNA of HDAC7, cross‐regulating each other's expression through miR‐1271‐5p. Moreover, both TTYH3 and HDAC7 3′UTRs possess biological functions in facilitating CRC progression.

In conclusion, the present study revealed that TTYH3 upregulation in CRC correlates with poor prognosis. We demonstrated that TTYH3 promotes CRC cell migration independent of its ion channel characteristics. Further investigation unveiled that TTYH3 competes with HDAC7 by sponging miR‐1271‐5p, consequently raising HDAC7 expression and promoting CRC metastasis as well as angiogenesis. These findings illuminated the regulation between TTYH3 and HDAC7 through ceRNA crosstalk in CRC and offer potential opportunities for developing novel therapy strategies.

## 4 MATERIALS AND METHODS

### 4.1 Human tissue samples

A total of 108 freshly removed cancer tissue samples, as well as 32 adjacent noncancerous colonic tissue samples, were obtained from the Department of Gastrointestinal Surgery, Xiangya Hospital (Central South University, Changsha, China). A total of 95 CRC paraffin tissue samples together with 52 adjacent normal colon paraffin tissues obtained from surgical resection patients were used in this study. Systematic clinical and follow‐up data were available from the Department of Pathology of Xiangya Hospital.

### 4.2 Cancer cell lines and culture

The human CRC cell lines HCT116, HT29, SW480, and SW620 and the human embryonic kidney cell line HEK‐293T were acquired from the American Type Culture Collection (ATCC). The HCT8, Caco2, RKO cell lines were supplied by Professor Wancai Yang (Institute of Precision Medicine, Jining Medical University).The normal colon epithelial cell line NCM460 was acquired from the Cell Bank of the Chinese Academy of Science (Shanghai, China). All CRC cells were cultured in RPMI‐1640 (BioInd, Beit Haemek, Israel) with 10% fetal bovine serum (BioInd) in an atmosphere of 5% CO_2_ at 37°C.

### 4.3 Plasmid, siRNA, and stable cell line construction and transfection

Plasmid encoding human TTYH3 was produced by PCR amplification and subcloned into pEGFP‐C1. HDAC7 plasmid (fused with Flag tag) was purchased from Addgene (USA). The wild‐type (WT) TTYH3‐3′UTR/HDAC7‐3′UTR was cloned into the psi‐CHECK™−2 vector. TTYH3‐3′UTR/HDAC7‐3′UTR mutant plasmids (MUT) with mutated target binding sites were created using the Mut Express‐II Fast Mutagenesis Kit (Vazyme, Nanjing, China). TTYH3 pore region mutant plasmid was subcloned into pCMV3 (SinoBiological, Beijing, China). All siRNAs were synthesized by RiboBio (Guangzhou, China) and used at a final concentration of 5 nM. Transfection was conducted with a jetPRIME kit (Polyplus Transfection, Illkirch, France) according to the manufacturer's instructions. For the stable cell line, TTYH3‐3′UTR, HDAC7‐3′UTR were cloned into pCDH‐CMV‐MCS‐EF1‐Puro (pCDH). Caco2 stable cell line expressing 3′UTR of TTYH3 or HDAC7 or empty plasmid was generated via retroviral infection. Briefly, The HEK‐293T cells were transfected with pCDH‐based 3′UTR or empty vector, pCDH:pMD2.G:pSPAX2 at the ratio of 3:1:2. The virus particles were collected 48 h after transfection. Caco2 cells were infected with recombinant lentivirus transducing units using 3 μg/mL polybrene (Sigma–Aldrich, St. Louis, MO), and the stable cell lines were maintained for 14 days with 3 μg/mL puromycin (MedChemExpress, Monmouth Junction, NJ). All stable cell lines were validated by qPCR and western blot (WB).

### 4.4 Bioinformatics analysis

Starbase version 2.0 (https://starbase.sysu.edu.cn/starbase2/index.php), 3.0 (http://starbase.sysu.edu.cn/index.php), and ceRDB (https://www.oncomir.umn.edu/cefinder/) were utilized for TTYH3 ceRNA prediction. Targetscan (https://www.targetscan.org/vert_80/) and Starbase were used to predict potential miRNAs that targeting TTYH3 and HDAC7. Genes positively correlated with TTYH3 or HDAC7 were obtained from the UALCAN database (https://ualcan.path.uab.edu/index.html).

### 4.5 Quantitative real‐time PCR

Total RNA was extracted using TRIZOL reagent (Invitrogen, Thermo Fisher Scientific, Waltham, MA), and RNA of proper integrity was reverse transcribed into cDNA using the GoScript Reverse Transcription System (Promega, Madison, WI). Real‐time quantitative PCR was conducted as previously described.^42^ The relative expression level was estimated by the 2^‒ΔΔct^ method. The results were normalized to GAPDH expression. Primer sequences were as follows: GAPDH (F: CTGGGCTACACTGAGCACC; R: AAGTGGTCGTTGAGGGCAATG); TTYH3 (F: TCCCCTTTTGGAGGAACACG; R: CCAGGAGGCAGATGATGACG); HDAC7 (F: GGCTGCTTTCAGGATAGTCG; R: GGTTCATCAGTTGCTGCGTC); E‐cadherin (F: CGCCATCGCTTACACCATCCTC; R: CTCTCTCGGTCCAGCCCAGTG); Vimentin (F: AGTCCACTGAGTACCGGAGAC; R: CATTTCACGCATCTGGCGTTC); N‐cadherin (F: TGCCATCATTGCCATCCTGCTC; R: CCCGGCGTTTCATCCATACCAC).

### 4.6 Western blot

WB was carried out as formerly described.^43^ The specific primary antibodies were as follows: HDAC7 (ABclonal; A7285, 1:1000), TTYH3 (Abcam; ab240580, 1:1000), GAPDH (Utibody; UM4002,1:5000), Vimentin (Proteintech; 10366‐1‐AP, 1:3000), E‐cadherin (Proteintech; 20874‐1‐AP, 1:3000), and N‐cadherin (Proteintech; 22018‐1‐AP, 1:2000).

### 4.7 Chemicals

NPPB was purchased from MCE (HY‐101012, USA), and dissolved in dimethyl sulfoxide. The final concentration of NPPB used in this study was 100 µM.

### 4.8 EdU and wound healing assay

The EdU (RiboBio) assay was performed according to the protocol as described. All the experiments were repeated at least three times. The numbers of positive cells were estimated using Image J (National Institutes of Health, USA).

For wound healing assay, cells were plated in a six‐well plate to form a monolayer a day before the assay. A micropipette tip scratched the well center uniformly and then put the cells at 37°C supplied with 5% CO_2_ for culture. Cell motility was assessed by measuring the rate of wound closure.

### 4.9 Migration assay

For transwell assay, a total of 3 × 10^5^ cells in 200 µL RPMI‐1640 were seeded into the top chamber of a 24‐well polycarbonate transwell filter (8 µm pore size; Corning Incorporated, USA), 800 µL media containing 20% FBS was placed in the lower chamber. After 24 h of incubation at 37°C and 5% CO_2_, cells were fixed with 4% paraformaldehyde and then stained with 0.5% crystal violet. The results were calculated by Image J.

### 4.10 Endothelial tube formation assay

HUVECs treated by different conditioned medium were cultured for 24 h. Then, cells were plated in 96‐well plates coated with 50 µL of Matrigel (BD Company, USA) at a concentration of 2 × 10^4^ cells per well. Tubules were photographed through microscopy and computed by ImageJ after incubating for 6 h at 37°C with 5% CO_2_.

### 4.11 Luciferase reporter assay

HEK‐293 cells were seeded in a 24‐well plate at 40−50% confluency and cotransfected miR‐1271‐5p mimics (or its negative control mNC) along with specific reporter plasmid. After 48 h, the luciferase assay was conducted using the Dual‐Glo Luciferase Kit (Promega) according to the instructions. Firefly luciferase activity in each sample was calculated by normalization to Renilla activity.

### 4.12 Animal study

Five‐week‐old male BALB/c (nu/nu) nude mice were used in the experiments performed according to the approved protocols. For subcutaneous xenograft generation, the mice were randomly divided into three groups (each group included six mice) and subcutaneously injected with 3 × 10^6^ cells per 100 µL (with stable Caco2‐VEC, Caco2‐TTYH3‐3′UTR, Caco2‐HDAC7‐3′UTR). Tumor volume (length × width^2^ × 0.5) was estimated every 2 days. After 5 weeks, all tumor grafts were excised, weighed, fixed in formalin, and embedded in paraffin, of which sections were stained with H&E and IHC.

For metastasis model was carried as follows: intra‐spleen injection with 3 × 10^6^ CRC cells (with stable VEC, TTYH3‐3′UTR, and HDAC7‐3′UTR) per 50 µL into the mice. The animals were excised after 6 weeks. Organs were isolated for histopathological examination of CRC tumors.

### 4.13 Immunohistochemistry

The protein expression of TTYH3 and HDAC7 were determined by IHC. The primary antibodies were as follows: anti‐TTYH3 (Thermo Fisher Scientific‐PA5‐62800), anti‐HDAC7(Bioss‐bs‐2890R, China), anti‐CD31(Servicebio, China). TTYH3 or HDAC7 staining were evaluated at high (×400) magnification in five fields. Tissue staining percentage was graded as: 1 (0−25%), 2 (26−50%), 3 (51−75%), or 4 (>75%); while cell intensity was scored as: 0 (negative), 1 (weak), 2 (moderate), or 3 (strong). Then, the total scores were calculated as staining percentage × intensity. Samples with Total score ≥6 were determined as high expression, and those with < 6 were low expression. The microvessel counting was done according to the method proposed by Weidner et al.^44^ First, the most intense area of tumor microvessel was identified by light microscopy. Then, individual microvascular counts of these most intense area in a 100× field was performed. Any CD31‐positive endothelial cell or cluster which clearly separated from adjacent cells was counted as a single microvascular. The highest number of microvessel in any 100× field was defined as MVD.

### 4.14 Statistical analysis

All quantitative data are shown as the mean ± SD or SEM and the experimental results were analyzed by SPSS 20.0 and GraphPad Prism 8.0 software. All assays were performed in triplicate. The statistical significance was assessed by *t*‐test, ANOVA, or log‐rank test. Statistical significance was defined as *p* < 0.05 (*p < 0.05, **p < 0.01, ***p < 0.001, ****p < 0.0001).

## AUTHOR CONTRIBUTIONS

Pengyan Lu, Shumin Deng, and Gang Yin conceived and designed the research studies. Pengyan Lu and Shumin Deng performed the experiments and generated the figures. Jiaxin Liu and Qing Xiao helped with the in vivo experiments and some vitro experiments. Shuojie Li helped finish the analysis of the results. Shumin Deng and Pengyan Lu wrote the paper together. Shuojie Li and Guang Shu contributed to check the figures and primary manuscript. Jiaxuan Xin contributed to the collection of samples. Zhengwei Zhou helped check and analyze the data. Gang Yin and Bo Yi supervised the project and revised the final manuscript. All authors read and approved the final manuscript.

## CONFLICT OF INTEREST STATEMENT

The authors declare no conflict of interest.

## ETHICS STATEMENT

*Animal*: The Institutional Animal Care and Use Committee of Central South University approved the protocols for animal care and euthanasia. All animals used in the experiments were performed according to the approved protocols (approval number: XMSB‐2022‐0190).

*Human samples*: The acquisition of fresh CRC tissues and paraffin tissue samples were approved by the Protection of Human Subjects Committee at Xiangya Hospital (approval number: 2022‐KT144), following the guidelines by the Declaration of Helsinki. Written informed consent was acquired from all participants.

## Supporting information

Supporting Information

## Data Availability

Four independent cohorts (GSE8671, GSE21510, GSE119409, and GSE10961) of CRC data were downloaded from the GEO database (http://www.ncbi.nlm.nih.gov/geo/). All data needed to evaluate the conclusions in the paper are present in the paper or in the Supplementary materials. Additional data are available upon reasonable request from corresponding author.
