# Supplementary material for: Tweety homolog 3 promotes colorectal cancer progression through mutual regulation of histone deacetylase 7
Source: MedComm (2020). 2024 May 31;5(6):e576. doi: 10.1002/mco2.576 (PMC11141500; doi:10.1002/mco2.576)
Supplement: Supplementary file 1 — Supporting Information [file MCO2-5-e576-s001.docx]

**Tweety homolog 3 promotes colorectal cancer progression through mutual regulation of histone deacetylase 7**

**Running title:** TTYH3 Promotes Colorectal Cancer Progression

Pengyan Lu^#1^, Shumin Deng^#1^, Jiaxin Liu^1^, Qing Xiao^3^, Zhengwei Zhou^1^, Shuojie Li^1^，Jiaxuan Xin^2^, Guang Shu^1^, Bo Yi^2*^, and Gang Yin^1,4,5*^

**Affiliations**

1 Department of Pathology, Xiangya Hospital, School of Basic Medical Sciences, Central South University, Changsha, China.

2 Department of Gastrointestinal Surgery, The Third Xiangya Hospital, Central South University, Changsha, China

3 Department of Pathology, The Third Xiangya Hospital, Central South University, Changsha, China.

4 National Clinical Research Center for Geriatric Disorders, Xiangya Hospital, Central South University, Changsha, China.

5 China-Africa Research Center of Infectious Diseases, School of Basic Medical Sciences, Central South University, Changsha, Hunan Province, China.

#These two authors: Pengyan Lu, Shumin Deng contributed equally.

***Corresponding auther: Bo Yi, Gang Yin**

Bo Yi

Department of Gastrointestinal Surgery, the Third Xiangya Hospital, Central South University, Changsha, 410013, China. phone: 86-0-13786179533; E-mail: yibo2018pro@126.com

Gang Yin

Department of Pathology, Xiangya Hospital, School of Basic Medical Sciences, Central South University, Changsha, 410013, China. phone: 86-0-18273182266; E-mail: [gangyin@csu.edu.cn](mailto:gangyin@csu.edu.cn)

**
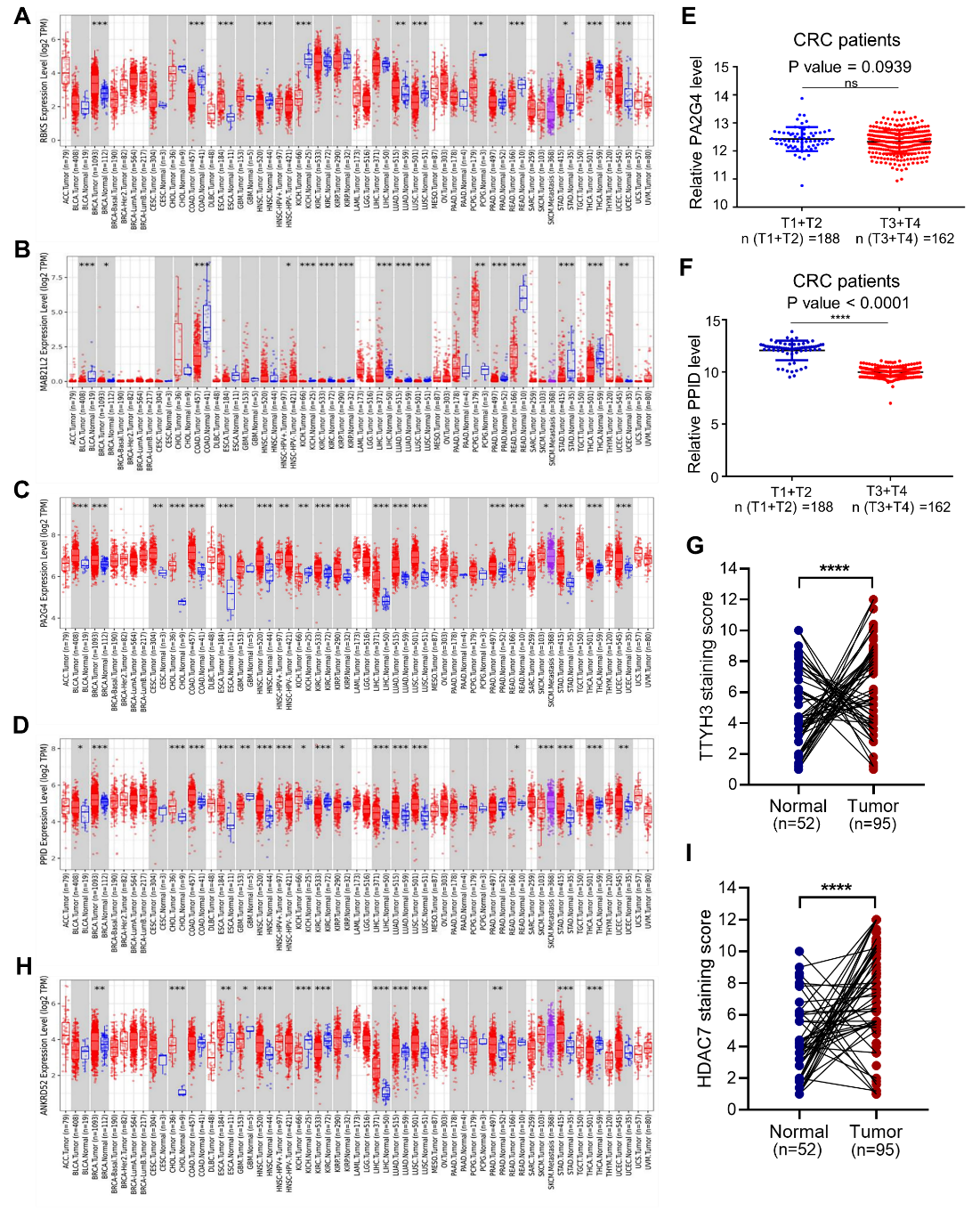
Supplementary Figure 1**

**A-D,H** Expression of RBK5(**A**), MAB21L2(**B**), PA2G4(**C**), PPID(**D**) and ANKRD52(**H**) in tumor and normal tissues. Red boxplot: tumor tissues; Blue boxplot: normal tissues; Purple boxplot: metastatic tissues. Data was obtained from TIMER database. **E, F** Analysis of the expression of PA2G4(**E**) and PPID(**F**) in different tumor stages of CRC samples. Data was downloaded from LinkedOmics dataset. **G, I** Quantification of TTYH3(**G**) and HDAC7(**I**) staining score in 52 matched adjacent normal colon and 95 CRC tissue samples. **P* < 0.05, ***P* < 0.01, ****P* < 0.001, *****P* < 0.0001.

**
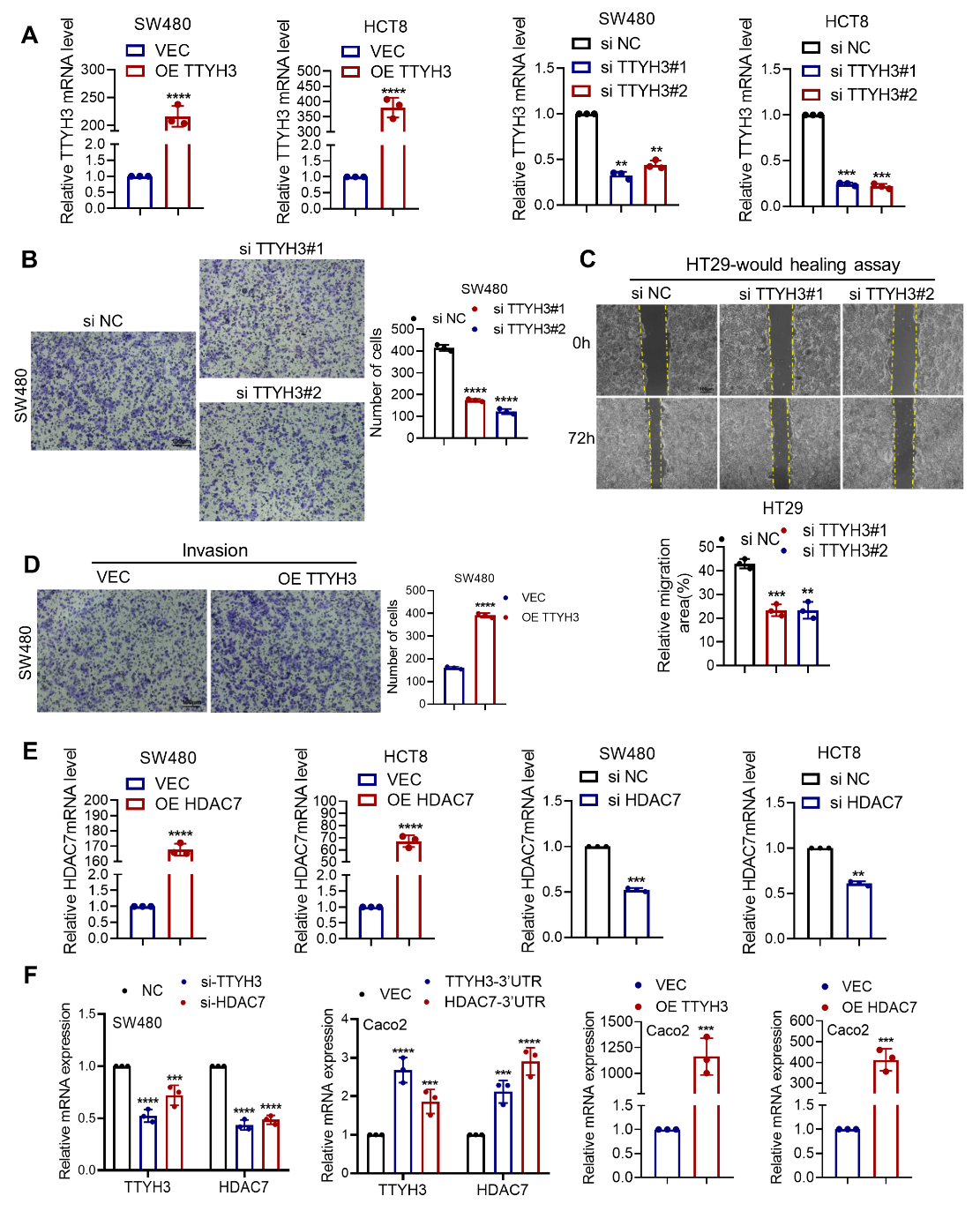
Supplementary Figure 2**

**A** Relative TTYH3 mRNA expression detected by qRT-PCR. **B-D** The cell motility detected by invasion assays(**B,D**) and wound healing assays(**C**). **E** Relative HDAC7 mRNA expression detected by qRT-PCR. **F** Relative TTYH3 and HDAC7 mRNA expression assessed by qRT-PCR. ***P* < 0.01, ****P* < 0.001, *****P* < 0.0001.

**
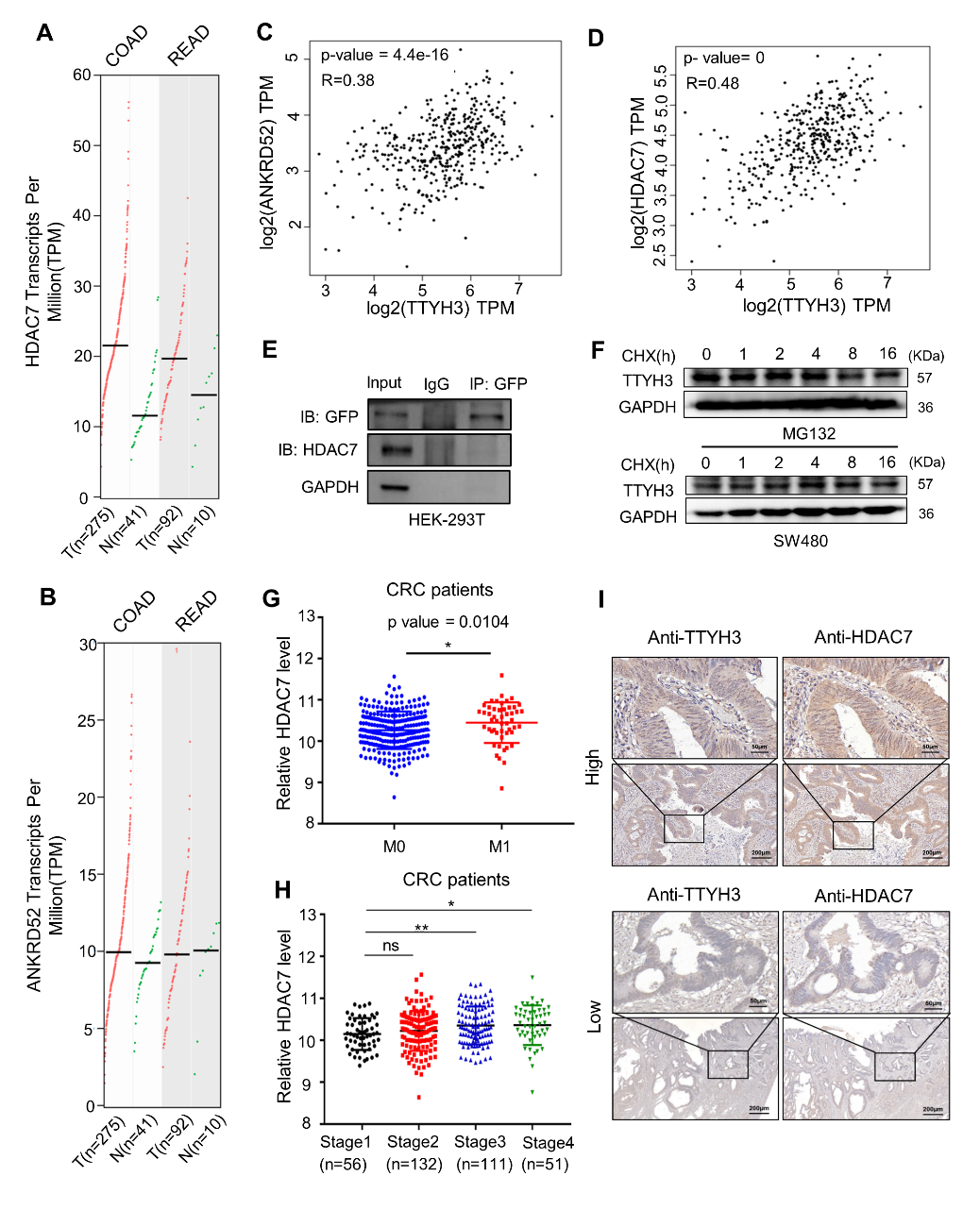
Supplementary Figure 3**

**A,B** Relative HDAC7(**A**) and ANKRD52(**B**) mRNA expression in GEPIA database **C,D** The correlation between TTYH3 and ANKRD52(**C**) or HDAC7(**D**) in CRC. Data was gained from GEPIA. **E** TTYH3 and HDAC7 interaction in HEK293T detected by Co-IP assays. **F** Relative TTYH3 and HDAC7 protein levels by WB. **G** CRC in situ tissue samples;m1: CRC distant metastasis tissue samples. Data was gained from LinkedOmics dataset. **G,H** Relative HDAC7 expression in metastasis or non-metastasis tissues(**G**) and in tumor tissues from different stages(**H**). M0: CRC in situ tissue samples; M1: CRC distant metastasis tissue samples. Data was gained from LinkedOmics dataset. **I** Relative TTYH3 and HDAC7 protein levels in CRC tissues by IHC. **P* < 0.05, ***P* < 0.01.

**
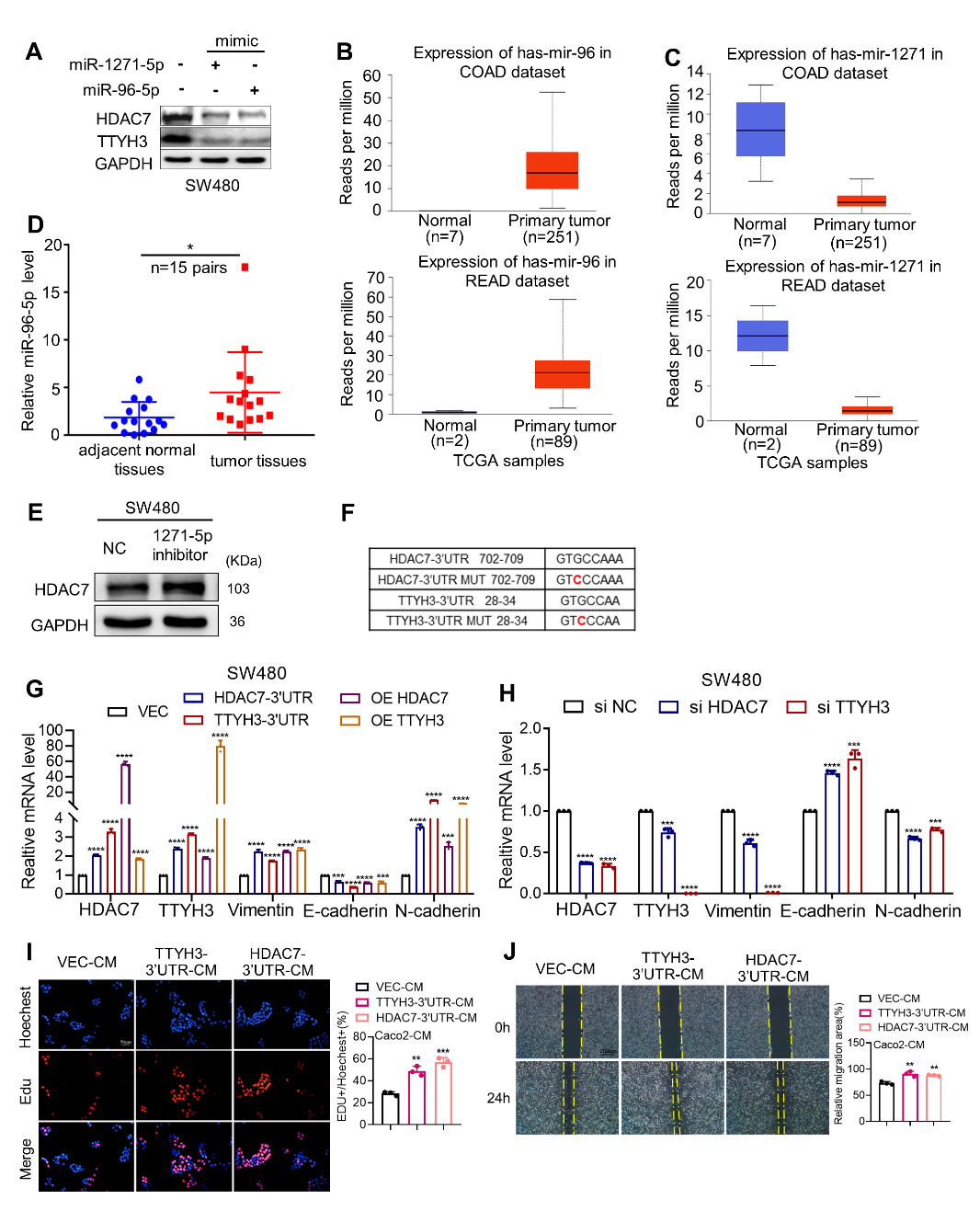
Supplementary Figure 4**

**A** Relative TTYH3 and HDAC7 protein levels were detected by WB after treatment with different mimics in SW480. **B,C** Relative miR-96-5p(**B**) and miR-1271-5p(**C**) expression in normal and primary tumor tissues. Data was gained from UALCAN database. **D** Relative miR-96-5p expression in adjacent normal and tumor tissues. **E** Relative HDAC7 protein levels after treatment with miR-1271-5p inhibitor in SW480. **F** The binding site of miR-1271-5p with HDAC7-3’UTR and TTYH3-3’UTR. The red letter means the mutant sites. **G,H** Relative mRNA expression assessed by qRT-PCR. **I** The HUVEC cell proliferation detected by EdU assay. Scale bar,50 µm. **J** The HUVEC cell motility examined by wound healing assay. Scale bar: 200 µm. **P* < 0.05, ***P* < 0.01, ****P* < 0.001, *****P* < 0.0001.
